# Application of the hierarchical model of intrinsic and extrinsic motivation in the context of exercise: a systematic review

**DOI:** 10.3389/fpsyg.2025.1512270

**Published:** 2025-02-13

**Authors:** Bernardo Viveiros, Miguel Jacinto, Raúl Antunes, Rui Matos, Nuno Amaro, Luís Cid, Nuno Couto, Diogo Monteiro

**Affiliations:** ^1^Polytechnic University of Leiria, Leiria, Portugal; ^2^CIDESD – Research Center in Sports Sciences, Health Sciences and Human Development, Vila Real, Portugal; ^3^Sport Sciences School of Rio Maior, Santarém Polytechnic University, Rio Maior, Portugal

**Keywords:** HMIEM, social factors, psychological needs, motivation, exercise

## Abstract

Given the increase in sedentary lifestyles and physical inactivity, various psychosocial approaches have been used to combat this epidemic. Several studies have used Self-Determination Theory (SDT) as a theoretical framework for studying behavioral change, as well as the Hierarchical Model of Intrinsic and Extrinsic Motivation (HMIEM) which, based on SDT, aims to explain how different levels of generality can be responsible for behavioral consequences. The aim was to investigate the associations between the variables that make up the HMIEM model applied to the context of physical exercise (gym exercisers). Following the PRISMA protocol and the PECOS strategy, the Web of Science, PubMed, Scopus, and SPORTDiscus databases were used to search for experimental and non-experimental studies written in English. Seven studies were considered for analysis and subjected to a methodological quality assessment The results showed that the variables that make up the social factors (e.g., supportive/thwarting behaviors) tend to be associated with satisfaction of basic psychological needs (BPN) (*r* = 0.51, *p* < 0.01; *r* = −0.73, *p* < 0.01) and with frustration of BPN (*r* = −0.39, *p* < 0.01; *r* = 0.78, *p* < 0. 01), BPN satisfaction and frustration tend to be associated with autonomous forms of motivation (*r* = 0.57, *p* < 0.01; *r* = −0.63, *p* < 0.01) and controlled forms of motivation (*r* = −0.76, *p* < 0.01; *r* = 0.46, *p* < 0.01) and autonomous and controlled forms of motivation are associated with behavioral consequences (e.g., intention) (*r* = 0.19, *p* < 0.01; *r* = −0.17, *p* < 0.01). This systematic review covers interpersonal behaviors and the bright and dark sides of SDT, showing that the positive alignment between the psychosocial determinants that make up the horizontal axis of the HMIEM is fundamental for adherence to and maintenance of sustainable physical exercise practices, and future studies should now address these issues in a longitudinal manner and perhaps move on to study the vertical axis of the HMIEM.

## 1 Introduction

According to the 2022 data provided by the World Health Organization (WHO), 27.5% of the global population does not adhere to the recommended levels of physical exercise for improving and safeguarding their health. In the European Union, 45% of respondents acknowledge never participating in any form of physical exercise, citing lack of time and motivation as the primary reasons for their sedentary behavior (Eurobarometer, 2022). It is well-established in the literature that physical exercise significantly contributes to both physical and mental well-being (Pedersen and Saltin, 2015; Warburton and Bredin, 2017). This behavior can contribute by reducing blood pressure (Dassanayake et al., 2022), improving body composition (Lopez et al., 2022; Mcleod et al., 2024), reducing anxious-depressive traits (Josefsson et al., 2014; Heissel et al., 2023) and increase quality of life (Marquez et al., 2020).

Gyms and health clubs are one of the population’s favorite places for exercise (International Health, Racquet and Sportsclub Association, 2020). However, several reports indicate losses of around 75% in the first 3 months of practice, falling to 50% when they reach 6 months of practice (IHRSA, 2020; Sperandei et al., 2016; IHRSA, 2023). As an example, Portugal has the highest sedentary lifestyle and physical inactivity rate in the European Union (73%) (Eurobarometer), with gym cancelation rates of 75% and retention rates of 25% (AGAP, 2022).

Based on Rodrigues et al. (2021), motivation is considered the most significant variable in explaining exercise participation or physical inactivity. This statement has been thoroughly tested across various contexts, including education (de Araujo Guerra Grangeia et al., 2016), physical education (Fernández-Espínola et al., 2020), sports (Monteiro et al., 2018), and exercise (Rodrigues et al., 2020). These studies share a common conceptual framework: the socio-cognitive macro theory known as Self-Determination Theory (SDT) (Deci and Ryan, 1985), which explores the impact of psychosocial determinants on behavioral outcomes in the exercise context (Ryan and Deci, 2000; Rodrigues et al., 2018). SDT encompasses six interconnected micro-theories that systematize key aspects of motivation, delineating the quality of motivation based on the degree of self-determination along a motivational continuum (Ryan and Deci, 2017). Each theory examines a specific motivational factor and integrates them to form the SDT, studying the types of motivation and mechanisms of self-determination (Deci and Ryan, 1985; Ryan and Deci, 2000).

The first micro theory of SDT is Cognitive Evaluation Theory (CET) (Deci and Ryan, 1985), which specifies the factors that account for variability in intrinsic motivation (Ryan and Deci, 2000). CET suggests that an individual’s cognitive evaluation of their environment can either facilitate or hinder intrinsic motivation by supporting or frustrating Basic Psychological Needs (BPN) (Ryan and Deci, 2000). According to SDT, people can exhibit six different forms of interpersonal behavior (Rocchi et al., 2017): Support for autonomy, Frustration of autonomy, Support for competence, Frustration of competence, Relationship support, and Relationship frustration. Empirical studies have indicated that supportive interpersonal relationships tend to be positively associated with BPN satisfaction (Rodrigues et al., 2021; Edmunds et al., 2007; Edmunds et al., 2008), whereas frustrating interpersonal behaviors tend to be linked with BPN frustration (Rodrigues et al., 2021; Ng et al., 2012).

The second micro theory proposed by SDT is the theory of basic psychological needs (TBPN) (Ryan and Deci, 2000), as outlined by Chen et al. (2015) and Ryan and Deci (2017). According to these scholars, BPN is considered innate and universal to all human beings, regardless of race, gender, and cultural background, and is directly linked to the regulation of motivation. The theory delineates three fundamental BPNs: the need for autonomy (the need for individuals to regulate their actions), the need for competence (the ability to interact effectively with their environment), and the need for relatedness (the ability to form interpersonal connections and interactions with others in their environment) (Ryan and Deci, 2000). From a theoretical perspective, the BPNs are distinct yet strongly connected and interdependent. When these needs are fulfilled, they are associated with more autonomous forms of motivation and play a pivotal role in the internalization and integration of behavior (Ryan and Deci, 2000; Ryan and Deci, 2019). Empirically, BPN satisfaction is strongly associated with self-determined motivation regulation (Rodrigues et al., 2022) but also with indicators of well-being (e.g., enjoyment) (Teixeira et al., 2018; Teixeira et al., 2021). The dark side of needs exists and must be considered. This concept is completely independent from the previous one and is mainly about the individual feeling that the context frustrates their BPN. This frustration may not only lead the individual to regulate their motivation in less self-determined ways, but it may also predict abandonment of physical exercise and indicators of ill-being (e.g., negative affect) (Rodrigues et al., 2021; Bartholomew et al., 2011; Gunnell et al., 2014; Vansteenkiste and Ryan, 2013).

The Organismic Integration Theory (OIT) (Deci and Ryan, 1985) is a theory that explores different forms of extrinsic motivation and the environmental factors that help or hinder the process of internalizing and integrating behavioral regulation (Ryan and Deci, 2000). The theory classifies motivation on a continuum, with its quality changing based on the level of self-determination (Rodrigues et al., 2022). This continuum can be understood from two perspectives: the macro perspective, which includes three forms of motivation: Amotivation (i.e., lack of intent to act), Extrinsic Motivation, and Intrinsic Motivation (i.e., pleasure and fun associated with the physical exercise) (Deci and Ryan, 1985; Vallerand and Toward, 1997), and the micro perspective, which further divides Extrinsic Motivation into four forms of regulation (Extrinsic Motivation, Introjected Motivation, Identified Motivation, and Integrated Motivation), in addition to Amotivation and Intrinsic Motivation (Deci and Ryan, 1985). As Teixeira et al. (2021) point out, external motivation is divided into two forms of behavior regulation of a more controlled nature: external regulation (i.e., carrying out the activity to avoid internal punishment), introjected regulation (i.e., carrying out the activity to avoid feelings of guilt and/or anxiety); and two of a more autonomous nature: identified regulation (i.e., the activity is identified as being important, even though they do not enjoy it) and integrated regulation (i.e., the activity is seen as an integral part of their life). It is expected that autonomous forms of motivation will have a positive association with behavioral consequences, while more controlled forms of motivation will be negatively associated with behavioral outcomes (Edmunds et al., 2008; Hagger et al., 2014; Heiestad et al., 2016; Ntoumanis et al., 2017).

### 1.1 Theorical framework

In line with SDT, Vallerand and Toward (1997) and Vallerand and Ratelle (2002) developed the Hierarchical Model of Intrinsic and Extrinsic Motivation (HMIEM) to explain how different levels of generality–global (personality), contextual (life domains), and situational (state)–influence an individual’s behavior. According to Vallerand and Lalande (2011), this model aims to organize and explain the mechanisms behind intrinsic and extrinsic motivation. The model is characterized by axes that determine it. On the vertical axis, we find the different levels of generality, and on the horizontal axis, we find psychosocial determinants. This arrangement leads to predictions regarding motivation and its emotional, cognitive, or behavioral outcomes. Some studies empirically support and analyze the motivational model, exploring both the positive and negative aspects of SDT in behavior consequences (Rodrigues et al., 2021; Rodrigues et al., 2022; Teixeira et al., 2018; Haerens et al., 2015; Rodrigues et al., 2019).

In addition to the HMIEM including the three micro theories of SDT, horizontally determining the psychosocial variables that impact behavioral consequences, this model is made up of relationships between its vertical axes, which are important to consider (Vallerand and Toward, 1997). At the top of the vertical axis, we can find the global level, referring to a person’s personality, in which motivation takes the form of general dispositions to engage in activities in an intrinsic or extrinsic way. The next level (contextual level) is represented under the specific contexts of life, considering the likelihood of individuals developing motivational orientations that may differ depending on the context, so we can consider this level to be quite volatile and prone to motivational variations (Vallerand and Toward, 1997; Núñez and León, 2019). Lastly, the situational level is considered by Vallerand (Vallerand and Toward, 1997) to be the most particular, referring to the exact moment when the behavior is carried out. The situational level can be considered as the state of motivation that an individual has when engaging in a specific action at a specific time. Thus, several studies assess the situational level in an attempt to understand the relationship between it and the higher levels (Vallerand and Toward, 1997; Núñez and León, 2019; Blanchard et al., 2003).

The three levels of generality are dynamically related to each other. In this way, the motivation of a particular level can influence the motivation of another hierarchical level. The occurrence of these relationships between levels is based on two distinct phenomena: the top-down effect and the bottom-up effect (Vallerand and Toward, 1997; Vallerand, 2007).

The top-down effect refers to the influence that higher levels of the hierarchy have on lower levels, i.e., global motivation influences contextual motivation and situational motivation. On the other hand, contextual motivation will influence situational motivation. The relationships established between each level are governed by the principle of proximity, i.e., each level will have a greater influence on the level immediately below it. In other words, overall motivation will have a greater influence on contextual motivation than on situational motivation (Vallerand and Toward, 1997; Vallerand, 2007) Much research has been carried out to support the top-down effect of HMIEM (Vallerand and Toward, 1997; Vallerand, 2007; Guay et al., 2003). For example, in the context of physical exercise, Ntoumanis and Blaymires (2003) demonstrated that situational motivation was positively predicted by contextual motivation measured 1 month in advance in physical exercise students.

The bottom-up effect is based on the same principle as its counterpart, but this time the authors of the model aim to explain that the lower levels can also influence the higher levels, i.e., situational motivation can influence contextual motivation and global motivation but based on the assumption of proximity, situational motivation will have a more significant influence on contextual motivation than on global motivation. These concepts have been empirically tested, demonstrating a positive and significant relationship between contextual and situational motivation, i.e., the greater and more self-determined the motivation to practice an activity (situational), the greater the motivation to practice that activity in general (contextual) (Guay et al., 2003; Ntoumanis and Blaymires, 2003; Gagné et al., 2003).

### 1.2 Current study

As mentioned above, human behavior is a process of inner reflection that can be influenced by various factors, such as interpersonal relationships and the contexts in which we live. Indeed, the social context can be a predictor of human behavior, since we extract all possible information to interpret our behavior (Rodrigues et al., 2018).

Only two systematic reviews to date in the context of exercise (Rodrigues et al., 2018; Teixeira et al., 2012) have analyzed parts of HMIEM based on SDT. The study by Teixeira et al. (2012) analyzed BPN satisfaction, motivation regulation and how this can predict different physiological outcomes (i.e., energy expenditure, body mass index), demonstrating a positive relationship between the more autonomous forms of motivation and physical exercise, in which identified regulation shows a tendency to predict short-term adoption and intrinsic motivation for long-term adherence. This study also shows that competence satisfaction and more intrinsic motives can positively predict adherence to exercise in a wide variety of samples and contexts. The study by Rodrigues et al. (2018) analyzed the associations between motivational variables (interpersonal behaviors, satisfaction/frustration of BPNs and motivation regulation) and behavioral outcomes (enjoyment, intention, persistence and adherence), showing that positive correlations are established between SDT and the various forms of behavioral expression in the context of physical exercise, and that these interventions are fundamental for promoting a continued practice of physical exercise.

In this sense, HMIEM has been applied in various contexts (i.e., education, sport) and in recent years there has been a notable growth in the application of this model in the context of physical exercise, although few studies have fully tested the sequence in this context or in other domains. In this way, the sequence bases its hypotheses on SDT to explain the organization between the constructs at different levels of generality and is a model that allows for a holistic understanding of motivation in different contexts, such as education, leisure and interpersonal relationships. In addition, this model aims to explain the changes in motivation regulation that occur in the individual over time, allowing the different types of regulation to be analyzed according to three levels of generality (Núñez and León, 2019).

To synthesize current literature of the HMIEM model applied to the context of physical exercise, we propose with this systematic review to investigate the associations of the variables that make up the motivational sequence (horizontal level/sequence) proposed by Vallerand (2007) (e.g., social factors, supportive/frustrating interpersonal behaviors) → BPN’s → (motivation regulation→ behavioral outcome) in exercise context (gym exercisers). In general, this systematic review adds to the empirical evidence of the six interpersonal behaviors (social factor) in relation to the other variables, considering the bright and dark sides of SDT and new behavioral variables (e.g., future behavior) in relation to the previous systematic reviews carried out.

## 2 Methodology

The systematic review was built following the items proposed by the PRISMA 2020 protocol (Page et al., 2021) and the methodology described by Bento (2014). After conceptualizing the systematic review, it was registered on the PROSPERO portal and assigned the registration number CRD42024559916 in 2024. The PECOS strategy (Morgan et al., 2018) was applied, helping to define the following parameters: (i) “P” (target population) corresponds to people who practice physical exercise, aged between 18 and 65, of any gender, ethnicity, or race; (ii) “E” (exposure) corresponds to people who practice physical exercise; (iii) “C” (comparison) not applicable; (iv) “O” (outcome) corresponds to the associations established between social factors, motivational variables and behavioral outcomes (e.g., pleasure, intention, and frequency); (v) “O” (outcome) corresponds to the associations established between social factors, motivational variables and behavioral outcomes (e.g., pleasure, intention, and frequency; pleasure, intention, and frequency); (v) “S” (study design) experimental and non-experimental studies (i.e., cross-sectional studies and longitudinal studies).

### 2.1 Information sources and research strategies

This study was carried out between April and 18 June 2024 by searching databases such as Web of Science, PubMed (all fields), Scopus, and SPORTDiscus (all fields), considering a time window from 1 January 1997 to 18 June 2024. The descriptors used in the search terms were: “Hierarchical Model of Intrinsic and Extrinsic Motivation ``, “motivational sequence ``, “instructor behaviors``, “social support``, “interpersonal behavio*``, “motiv*``, “basic psychological needs``, “enjoyment,” “intention,” “frequency,” “health clubs,” “gym,” “fitness” and “exercise,” using the Boolean operators “AND” and “OR,” as shown in Table 1.

**Table 1:** Research strategy.

Investigator number	Descriptors
1	(“Hierarchical Model of Intrinsic and Extrinsic Motivation” OR “motivational sequence”) AND (exercise OR gym OR fitness OR “health clubs”) AND (interpersonal behavio* OR “instructor behaviors” OR “social support” OR motiv* OR “behavior regulation” OR “basic psychological needs” OR enjoyment OR frequency OR intention OR persistence)

### 2.2 Eligibility criteria

The PECOS strategy (Morgan et al., 2018) was applied, helping to define the following parameters: (i) “P” (target population) corresponds to people who practice physical exercise, aged between 18 and 65, of any gender, ethnicity, or race; (ii) “E” (exposure) corresponds to people who practice physical exercise; (iii) “C” (comparison) not applicable; (iv) “O” (outcome) corresponds to the associations established between social factors, motivational variables and behavioral outcomes (e.g., pleasure, intention, and frequency); (v) “O” (outcome) corresponds to the associations established between social factors, motivational variables and behavioral outcomes (e.g., pleasure, intention, and frequency; pleasure, intention, and frequency); (v) “S” (study design) experimental and non-experimental studies (i.e., cross-sectional studies and longitudinal studies).

To select the studies, the following inclusion criteria were considered: (i) experimental and non-experimental studies (i.e., cross-sectional studies and longitudinal studies); (ii) the studies had to be published between 1 January 1997 (corresponding to the year Vallerand published the HMIEM model) and 18 June 2024; (iii) they had to be written in English; (iv) the studies had to analyze parts (at least 2 variables of the model) or all of the HMIEM model; (v) the exercise participants had to be aged between 18 and 65; (vi) healthy individuals.

Similarly, exclusion criteria were drawn up and taken into consideration: (i) studies that included sports practitioners would not be selected as sport and exercise are different concepts (Caspersen et al., 1985); (ii) practitioners should not be younger than 18 years old or older than 65 years old; (iii) studies published in the field of physical education would also be excluded as this type of physical activity is different from regular exercise (Caspersen et al., 1985); (iv) gray literature would not be included for analysis and (v) systematic reviews.

### 2.3 Data extraction process

The study was carried out independently by two researchers, who downloaded all the studies from the databases into the ENDNOTE X7 software and eliminated duplicate articles. Initially, the articles were excluded by reading the titles and abstracts. In a second phase, the articles were read in full and those that did not fulfill the previously established eligibility criteria were excluded, leaving only 7 articles. The results of all the phases were compared (BV and MJ) discrepancies between the researchers were resolved by a third researcher (DM) One of the researchers (BV) supervised by other two (MJ and DM) exported the relevant information from the articles and entered it into Table 2 (authors, year of publication, continent, parents, objectives, participants, study design, measures used to assess the variables, results, variables under study and methodological quality).

**Table 2:** Characteristics of the selected studies for analysis.

Studies	Aims	Participants/Age	Design	Assessment instruments/technique	Main conclusions	Variables in study	Methodology quality
Edmunds et al. (2008)	(1) To analyze whether the teaching style of an exercise instructor can be manipulated in such a way that it satisfies more autonomy support, structure, and interpersonal involvement. (2) To analyze the impact of an exercise class taught according to socio-contextual variables on psychological needs, autonomous motivation and behavior; (3) To analyze the motivational sequence incorporated into SDT.	Exercisers (*N* = 56) Ages ranged from 18–53 in the experimental group (*M* = 21.26 years, SD = 3.80) and from 18–38 in the control group (*M* = 21.36 years, SD = 6.71).	Experimental (10 weeks)	Perceived Environmental Supportiveness Scale (PESS); Psychological Need Satisfaction Scale (PNSS); Behavioral Regulation Exercise Questionnaire 2 (BREQ-2) + Exercise Motivation Scale (EMS); Positive Affect and Negative Affect Scale (PANAS); Adherence (register); Intention.	Control group: There was a significant decrease in autonomy support, an increase in amotivation, and a decrease in behavioral intention over time. In addition, there was a significant increase in perceived competence and introjected regulation. Experimental group: This group showed a significantly greater linear increase in structure, interpersonal involvement, fulfillment of relationship and competence needs, and positive affect. Attendance rates were also significantly higher in the experimental group	Perception of autonomy support, structure, and interpersonal involvement; BPN, regulation of motivation (intrinsic, integrated, introjected, amotivation), Adherence, Intention, and positive and negative affect.	17
Ng et al. (2013)	This study aimed to analyze how others can support or counteract the psychological needs of exercisers with weight control goals, and how this condition can impact well-being and behaviour.	Exercisers (*N* = 156) Average age of participants (*M* = 31.01 years, SD = 13.21)	Retrospective (6 months)	Health Care Climate Questionnaire (HCCQ) + Controlling Coach Behaviors Scale (CCBS); Basic Needs Satisfaction in Sport Scale (BNSE) + Psychological Need Thwarting Scale (PNTS); GLTEQ; Eating Behaviors; Life Satisfaction; Self-esteem; Hospital Anxiety and Depression Scale (HADS).	The perception of autonomy support from others was associated with the satisfaction of psychological needs, while controlling behaviors were linked to the frustration of these needs. The satisfaction of needs was positively related to greater satisfaction with life, while the frustration of needs was related to negative consequences, such as depressive symptoms and unhealthy eating behaviors.	Perception of autonomy support/control; Satisfaction and Frustration of BPN; Exercise behaviors; Healthy and unhealthy eating behaviors; Psychological well-being/ill-being;	16
Ng et al. (2013)	The study aimed to analyze how the perception of autonomy support/control from others can predict BPNs, motivation regulation, and weight control behaviors (i.e., physical exercise and eating habits).	235 Practitioners trying to control their weight (183 F; 156 M) Ages ranged from 18 to 64 (*M* = 27.39 years, SD = 8.96)	Cross-sectional	Perception of autonomy support + CCBS; Basic Needs Satisfaction in Sport Scale (BNSE) + Psychological Need Thwarting Scale (PNTS); BREQ-2; GLTEQ; Eating Behaviors.	The results showed that when others supported perceived autonomy, participants reported more autonomous levels of motivation for weight management, which in turn predicted greater physical activity and healthy eating behaviors. In contrast, when others adopted controlling behaviors over autonomy, participants reported more controlled forms of motivation, predicting lower physical activity and healthy eating behaviors, as well as a greater preponderance of unhealthy eating behaviors.	Perception of autonomy support/control; BPN satisfaction and frustration; Exercise behaviors; Healthy and unhealthy eating behaviors	12
Jones et al. (2017)	This study aimed to explore two psychological characteristics that are potential predictors of people’s reactions to exercise classes: attention style and contextual motivation	Female exercisers (*N* = 417) Average age of participants (*M* = 37.2 years, SD = 13.7)	Cross Sectional	Attentional Focus Questionnaire (AFQ) + Cognitive Index (CI); EMS; Affect Grid (AG); Flow State Scale-2 (FSS-2); Behavioral Intent.	Attention style (internal focus) proved to be significant (*p* < 0.05) for affective, cognitive and behavioral results. Practitioners with an internal focus proved to be more self-determined and obtained more positive results than those with an external focus. Highly self-determined individuals obtained better results in the behavioral, cognitive, and affective variables. Almost 29% of the variation in the participants’ affective valence can be explained by the behavioral regulations of the externally focused practitioners.	Attention Style; Motivation Regulation; Affective Valence; Intention; Concentration and Excitement	12
Rodrigues et al. (2019)	Based on the theory of self-determination, the study aimed to analyze the Bright and Dark sides of motivation as predictors of enjoyment, intention, and persistence in physical exercise.	575 Portuguese exercisers (230 F) Ages ranged from 18–65 (*M* = 34.07 years, SD = 11.47)	Cross Sectional	Interpersonal Behavior Questionnaire (IBQ); Basic Psychological Need Satisfactions and Frustration Scale Portuguese version (BPNSFS-E); BREQ-3; Physical Activity Enjoyment Scale (PACES); Intention; Persistence.	Positive and significant associations were found between the “Bright” side of motivation and the predictors under study. However, the “Dark” side showed negative and significant correlation values with all the predictors. Through a mediation analysis, the “Bright” side model showed total mediation, with the influence of the mediators always being significant in the model. The “Dark” side model showed no significant direct or indirect effects on persistence. Intention had a significant effect on exercise persistence but only explained 11 percent of the variance. The overall structural model explained 14 percent of the variance, considering all the direct and indirect effects.	Interpersonal behaviors of support and thwarting; BPN Satisfaction and Frustration; Regulation of motivation; Enjoyment; Intention; Persistence.	16
Rodrigues et al. (2021)	(1) Considering the motivational sequence proposed by SDT, this study aimed to test the effect of past behavior on future behavior.	293 Portuguese exercisers (166 F; 127 M) Ages ranged from 18 to 65 (*M* = 36.57 years, SD = 11.25)	Cross Sectional	IBQ; BPNSFS-E; BREQ-3; PACES; Intention and Past and future behavior (frequency recording)	All the variables under study correlated positively and significantly with each other, except for the relationship between past and future behaviors with interpersonal support behaviors and the satisfaction of basic psychological needs. The results show positive associations between all the constructs, respecting the motivational sequence. However, with the addition of past behavior, the model cancels out the significance between intention and future behavior, and past behavior becomes the significant mediator between this relationship. This also happens with the indirect effects of the model: when past behavior is added to the model, it eliminates all the indirect effects of the constructs on future behavior.	Interpersonal support behaviors; BPN satisfaction; Motivation regulation (autonomous regulation); Pleasure; Intention; Past and future behavior	16
Cho et al. (2023)	This study aimed to analyze the impact of social support from family and friends on the intention to practice physical exercise, using BPNs, intrinsic motivation, and attitude as mediators.	Exercising university students in Singapore (*n* = 318) Ages ranged from 18 - >40	Cross Sectional	Social Support for Exercise (SSE); Basic Psychological Needs in Exercise Scale (BPNES); BREQ-2; Attitude e Intention.	The results showed a direct effect between the social support provided by friends on BPNs, and that needs had an indirect effect on the intention to exercise, through the mediation of intrinsic motivation and attitude.	Family and Friends’ Social Support; BPN’s; Intrinsic Motivation; Attitude and Intention	

### 2.4 Methodological quality assessment

Downs and Black (1998) were used to analyze the methodological content of the studies. This instrument consists of 27 questions that seek to determine the quality of the study considering various parameters, namely the design of the study, the adequacy of the statistical procedures, the clarity of the description, and the main conclusions. The scale’s score ranges were given corresponding quality levels: excellent (26–28); good (20–25); fair (15–19); and poor (≤14). No items were removed from the scale, leaving the 28 possible points. The methodological quality of the studies was measured independently by two reviewers (BV and MJ), and they were compared and discussed to reach a consensus. The scores awarded to each study are shown in Table 2.

## 3 Results

### 3.1 Selection of studies

**Figure A1:**
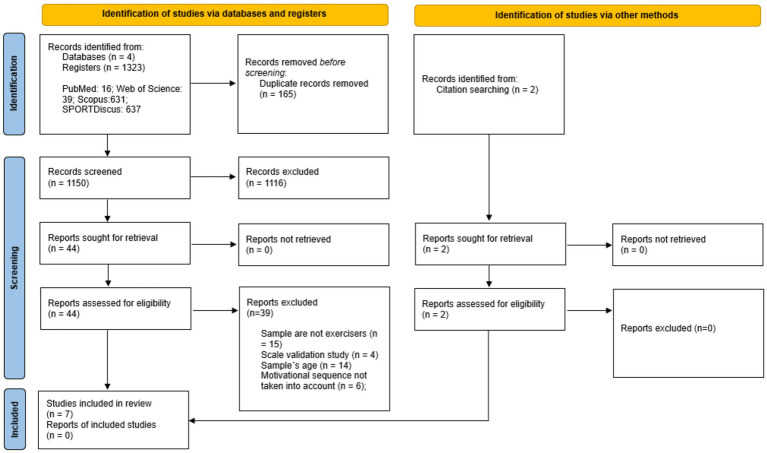
PRISMA flow diagram illustrating each phase of the search and selecting process.

After searching various databases, 1323 studies were identified. In the first phase, duplicate articles were eliminated by reading the titles and abstracts (i.e., articles that did not correspond to scientific publications and did not have a well-established and objective experimental design). After this stage, 44 studies with relevant potential for the study were identified and moved on to the next stage. Considering the established eligibility criteria and the reading of the articles, a sample of 7 studies was drawn up for analysis (5 studies were chosen by searching databases and registers, while the remaining 2 were obtained by other search methods, namely citation searches) (Table 2). Concerning the eliminated studies, 15 studies were considered eligible due to their sample not being physical exercise practitioners, 4 studies were related to the validation of scales/questionnaires and 6 studies did not include the motivational sequence. The PRISMA flow chart illustrating each phase of the search and selection process is presented in Figure A1.

### 3.2 Origin

Most of the studies (86%) were carried out on the European continent, specifically in Portugal (Rodrigues et al., 2020; Rodrigues et al., 2022) and the United Kingdom (Edmunds et al., 2008; Ng et al., 2013; Jones et al., 2017; Ng et al., 2014). The only study carried out outside the European continent was by Cho et al. (2023), conducted in Singapore, on the Asian continent.

### 3.3 Participants

The study samples were made up of exercisers with a wide age range (between 18 and 65 years old). In total, this systematic review includes 2023 healthy exercisers, with females predominating (*N* = 1399). The study with the largest number of participants was by Rodrigues et al. (2020) with 575 participants (female gender = 230). The sample size was calculated through the G*Power software using the following parameters: predicted effect size of *f*^2^ = 0.01, *α* = 0.05, and statistical power = 0.95.

### 3.4 Measuring instruments/techniques

Throughout the seven studies analyzed, various measuring instruments were identified to assess the horizontal axis of the HMIEM, made up of: social factors, mediators (basic psychological needs - BPN’s), motivation and consequences.

In this systematic review we found 5 assessment tools for social factors, such as the Interperssonal Behavior Questionnaire: (IBQ) (Rodrigues et al., 2020): this instrument assesses supportive and frustrating behaviors. The scale consists of 24 items (4 items per factor) in which it measures autonomy support (item 1: “My exercise instructor supports my choices”), competence (item 9: “My exercise instructor encourages me to improve my skills”) and relationship (item 5: “My exercise instructor really enjoys spending time with me”), as well as autonomy frustration (item 8: My exercise instructor imposes their opinions”), competence (item 22: “My exercise instructor questions my ability to overcome challenges”) and rapport (item 24: My exercise instructor does not empathize with me”), taking into account the practitioners’ perception of the fitness instructors’ behavior during their activities in the gym. The type of response used in this scale is Likert-type, scored between 1-"do not agree” and 7-"totally agree.” The results of this instrument support its nomological validation [χ2 = 3212.946 (1014); χ2/df = 3.16; B-*p* < 0.001, TLI = 0.905, CFI = 0.915, SRMR = 0.048, RMSEA = 0.051 (90%CI = 0.049, 0.054)].

For the evaluation of BPN, we found 4 evaluation instruments, with the following standing out Basic Psychological Need Satisfactions and Frustration Scale Portuguese version (BPNSFS-E) (Rodrigues et al., 2022): this instrument is used to measure autonomy satisfaction (item 1: “I feel a sense of choice and freedom in the exercises I do”), competence (item 5: “I feel confident that I can do the exercises correctly”) and relatedness (item 9: “I feel connected to the people at the gym who care about me and for whom I care”). This scale also assesses the frustration of autonomy (item 8: “I feel forced to do exercises that I would not choose to do”), competence (item 12: “In some exercises, I feel disappointed with my performance”) and relatedness (item 22: “I feel that the relationships I have at the gym are only superficial”). This instrument had results that support its nomological validation [χ2 = 471.814 (237); χ2 /df = 1.99; B-p < 0.001, TLI = 0.940, CFI = 0.948, SRMR = 0.038, RMSEA = 0.047 (90%CI = 0.042, 0.052)].

To assess how motivation is regulated, we found 3 evaluation instruments, in which we highlight the Behavioral Regulation Exercise Questionnaire 3 (BREQ-3) (Cid et al., 2018): This questionnaire measures the six behavioral regulations according to the SDT motivational continuum. This version has 18 items measuring amotivation (item 1: “I do not see why I have to exercise”), external (item 14: “I exercise because others will be unhappy with me if I do not”), introjected (item 3: “I exercise because I feel guilty when I fail a training session”), identified (item 16: “I exercise because I value the benefits of exercise”), integrated (item 5: “I exercise because it is related to my life goals”) and intrinsic (item 12: “I exercise because I enjoy my training sessions”). The type of response is a 5-point scale ranging from 0- “totally disagree” to 4- “totally agree,” on how exercisers perceive their motivation to practice physical exercise. The results of this instrument support its nomological validation (χ2 = 254.08; df = 120; B-S *p* < 0.001; SRMR = 0.04; NNFI = 0.93; CFI = 0.95; RMSEA = 0.06; 90% CI = 0.05–0.06).

In conclusion, we found 15 behavioral consequence instruments, with Ajzen (1985) recommendations standing out as one of the most widely used to measure the intention to exercise.

### 3.5 Study results

#### 3.5.1 Social factors → BPN

All the studies analyzed are at the contextual level according to the model recommended by Vallerand and Toward (1997). Thus, the social support provided by friends had a direct effect on BPNs(*β* = 0.496, SE = 0.042, z = 7.87) (Cho et al., 2023). Similarly, it has been shown that the perception of autonomy can positively predict satisfaction with BPNs (*r* = 0.38, *p* < 0.01) and negatively predict frustration with BPNs (*r* = −0.19, *p* < 0.01), while control behaviors correlate negatively with satisfaction with BPNs (*r* = −0.19, *p* < 0.01) and positively with frustration with BPNs (*r* = 0.41, *p* < 0.01) (Ng et al., 2013; Ng et al., 2014). However, Rodrigues et al. (2020) shows a positive and significant relationship between supportive behaviors and the satisfaction of BPNs (*r* = 0.51, *p* < 0.01) and a negative relationship between these behaviors and the frustration of BPNs (*r* = −0.39, *p* < 0.01), this trend reversing when the perception of frustration behaviors is the independent variable (*r* = −0.73, *p* < 0.01; *r* = 0.78, *p* < 0.01, respectively), but only positive and significant predictive values were reported between the perception of frustration behaviors and BPN frustration (*β* = 0.684, *R*^2^ = 0.467, CI 95% = 0.328/0.797, *p* = 0.002). However, Rodrigues et al. (2022) again demonstrated a positive and significant relationship between the perception of supportive behaviors and the satisfaction of LBWs (*r* = 0.64, *p* < 0.01), but with the independent variable showing positive and significant predictive values with the dependent variable, with (*β* = 0.67, *R*^2^ = 0.46, CI 95% = 0.54/0.78, *p* = 0.001) and without (*β* = 0.66, *R*^2^ = 0.46, CI 95% = 0.54/0.78, *p* = 0.001) past behavior integrated into the model.

#### 3.5.2 BPN → regulation of motivation

BPN satisfaction was positively and significantly associated with more autonomous forms of motivation, with correlation values ranging from 0.40 to 0.63 with *p* < 0.01 (Rodrigues et al., 2020; Rodrigues et al., 2022; Ng et al., 2014). However, a negative and significant relationship was demonstrated between satisfaction with BPNs and controlled forms of motivation regulation, with correlation values ranging from –0.26 to –0.76 with *p* < 0.01 (Rodrigues et al., 2020; Ng et al., 2014). BPN frustration is positively and significantly related to controlled forms of motivation regulation (e.g., *r* = 0.46, *p* < 0.01; Rodrigues et al., 2020) and negatively and significantly related to more autonomous forms of motivation (e.g., *r* = −0.63, *p* < 0.01; Rodrigues et al., 2020). Still about the relationship between BPNs and forms of motivation regulation, it was shown that at the start of a 10-week intervention program, competence (*β* = 0.16, *p* < 0.01) could be a positive predictor of integrated motivation and autonomy (*β* = 0.39, *p* < 0.001) a negative predictor of identified regulation. Over time, the effects of need for autonomy and autonomy support may vary on identified regulation (need for autonomy *β* = 1.06, *p* < 0.001; autonomy support *β* = 0.48, *p* < 0.01) and intrinsic motivation (need for autonomy *β* = 0.64, *p* < 0.01; autonomy support *β* = 0.62, *p* < 0.01) (Edmunds et al., 2008).

#### 3.5.3 Regulation of motivation → consequences

In the studies analyzed, more autonomous forms of motivation regulation correlated positively and significantly with the various behavioral consequences. In the study by Rodrigues et al. (2020), there was a positive and significant correlation between forms of autonomous regulation and enjoyment (*r* = 0.70, *p* < 0.01), intention (*r* = 0.19, *p* < 0.01) and persistence (*r* = 0.67, *p* < 0.01). It was also found that forms of controlled regulation correlated negatively and significantly with enjoyment (*r* = −0.48, *p* < 0.01), intention (*r* = −0.17, *p* < 0.01) and persistence (*r* = −0.51, *p* < 0.01). In a second study published by the same author, it was found that in addition to the forms of autonomous regulation continuing to show positive and significant correlation values with enjoyment (*r* = 0.68, *p* < 0.01) and intention (*r* = 0.42, *p* < 0.01), they also correlated with past behavior (*r* = 0.27, *p* < 0.01) and future behavior (*r* = 0.28, *p* < 0.01). In the study by Ng et al. (2013), the tendency for more autonomous forms of motivation to correlate positively and significantly with physical exercise frequency was maintained (*r* = 0.18, *p* < 0.01), but controlled motivation (*r* = −0.09) and amotivation (*r* = −0.04) correlated negatively with this variable, with no significant values. In conclusion, Edmunds et al. (2008) show that amotivation is a negative predictor of positive affect (*β* = 1.06, *p* < 0.01) and a positive predictor of negative affect (*β* = 0.26, *p* < 0.05), as is external regulation (*β* = 0.43, *p* < 0.01), while integrated regulation positively predicts positive affect (*β* = 0.30, *p* < 0.05).

#### 3.5.4 Indirect effects

Regarding the indirect effects of social support variables, it has been shown that autonomy support has a significant indirect effect on the frequency of physical exercise (*β* = 0.06, *p* < 0.01) (Ng et al., 2014). Similarly, the perception of control behaviors seems to have a significant indirect effect on autonomous motivation (*β* = −0.234, *R*^2^ = 0.054, CI 95% = −0.356/−0.085, *p* = 0.019), controlled motivation (*β* = 0.644, *R*^2^ = 0.414, CI 95% = 0.352/0.684, *p* = 0.002) and depressive symptoms (*β* = 0.09, CI 95% = 0.00/ 0.21) (Rodrigues et al., 2020; Ng et al., 2013). The perception of supportive behavior seems to have a significant indirect effect on future behavior (*β* = 0.03, *R*^2^ = 0.00, CI 95% = 0.02/0.06, *p* = 0.001), but when past behavior is included in the model the effect becomes non-significant (*β* = 0.00, *R*^2^ = 0.00, CI 95% = −0.01/0.01, *p* = 0.135) (Rodrigues et al., 2022). Social support from friends also had significant indirect effects (*p* < 0.001) on intrinsic motivation (*β* = 0.299, SE = 0.044, *z* = 6.80), attitude (*β* = 0.117, SE = 0.030, z = 3.87) and intention (*β* = 0.150, SE = 0.040, *z* = 3.79). BPN satisfaction also seems to have an indirect effect on behavioral consequences, namely enjoyment (*β* = 0.52, *R*^2^ = 0.27, CI 95% = 0.38/0.64, *p* = 0.001), intention (*β* = 0.21, *R*^2^ = 0.04, CI 95% = 0.14/0.30, *p* = 0.001), persistence (*β* = 0.047, *R*^2^ = 0.002, CI 95% = 0.028/0.070, *p* = 0.001) and future behavior (*β* = 0.05, *R*^2^ = 0.00, CI 95% = 0.02/0.08, *p* = 0.001), but when past behavior is included in the model as a mediator, the indirect effect becomes non-significant (*β* = 0.01, *R*^2^ = 0.00, CI 95% = −0.01/0.02, *p* = 0.143) (Rodrigues et al., 2020; Rodrigues et al., 2022). Intrinsic motivation, in turn, seems to have a significant indirect effect on intention (*β* = 0.501, SE = 0.125, z = 4.01) (Cho et al., 2023).

#### 3.5.5 Quality of the studies

The methodological quality of the studies was assessed as ranging from poor to excellent. The studies analyzed had different ratings, ranging from 11 (poor) to 17 (fair), as can be seen in Table 3. Although the Downs and Black scale (Downs and Black, 1998) has been validated to assess the methodological quality of non-randomized studies (i.e., cross-sectional studies), it has questions aimed at randomized studies (i.e., experimental studies), To illustrate, the absence of mandatory blinding of respondents and evaluators in practically all cross-sectional studies - as most apply questionnaires or scales, often after a prior anchoring/adaptation process - results in a score of 0 on items 14 and 15 of the Downs and Black scale (Downs and Black, 1998) (“Was an attempt made to blind the study subjects to the intervention they received?” and “Was there an attempt to blind the people measuring the main results of the intervention?”).

**Table 3:** Assessment of the methodological quality of the studies.

Study	Score	Methodological quality
Edmunds et al. (2008)	17	Fair
Ng et al. (2013)	16	Fair
Ng et al. (2013)	12	Poor
Jones et al. (2017)	12	Poor
Rodrigues et al. (2019)	16	Fair
Rodrigues et al. (2021)	16	Fair
Cho et al. (2023)	11	Poor

The score ranges of the scale were given corresponding quality levels: excellent (26–28); good (20–25); fair (15–19); and poor (≤14).

## 4 Discussion

This systematic review aimed to analyze the literature that applies the motivational sequence based on SDT and HMIEM. Associations between variables in the gym/fitness context were examined, including social factors (e.g., supportive or frustrating interpersonal behaviors), BPN, motivation regulation, and behavioral outcomes. The hierarchical model (Vallerand and Ratelle, 2002; Vallerand, 2007) offers a multi-level perspective on human motivation, organizing and simplifying the implicit mechanisms of the different forms of motivation regulation (Vallerand and Lalande, 2011). In this way, the model specifies the types of motivation, organizing them into different levels of analysis, demonstrating that they are shaped by social and personal determinants and generate behavioral, affective, or emotional results.

### 4.1 Social factors

According to this systematic review, all variables that support behavior (e.g., supportive behavior by exercise instructors) tend to result in higher levels of BPN satisfaction and lower levels of BPN frustration (Rodrigues et al., 2020; Edmunds et al., 2007; Rodrigues et al., 2022; Ng et al., 2013; Murcia and Sánchez-Latorre, 2016). The opposite is true when these behaviors (support) are controlling, tending to lower levels of BPN satisfaction and increase levels of BPN frustration on the part of practitioners (Rodrigues et al., 2020; Rodrigues et al., 2022; Ng et al., 2013; Murcia and Sánchez-Latorre, 2016; Rocchi and Pelletier, 2018). Social support offered by third parties, such as friends, also showed a positive association with BPNs, as evidenced by Cho et al. (2023). In contrast, the study by Murcia and Sánchez-Latorre (2016) showed that family support maintains a positive and significant association with basic psychological needs, exerting a direct effect on them. The discrepancy between the results of these studies can be attributed to differences in the samples used, as well as variations in age groups and recruitment conditions, which may influence the results obtained (Cho et al., 2023). Significant relationships tend to exist between autonomy support and the frequency of physical exercise (Ng et al., 2013), i.e., exercisers tend to exercise more often and sometimes at higher levels of intensity (Murcia and Sánchez-Latorre, 2016), and there tend to be relationships between perceptions of control in autonomous and controlled motivation (Rodrigues et al., 2020) and in depressive symptoms (Ng et al., 2013).

The empirical relationships discussed in this section can be substantiated from a conceptual perspective. Firstly, the results showing the interaction between variables that support or frustrate BPN behavior and satisfaction are in line with the theoretical assumptions of CET. This micro-model of SDT postulates that external events, such as supportive behavior on the part of instructors, family, and friends, can facilitate or hinder the internalization of behavior (Ryan and Deci, 2000). The context in which practitioners are placed, such as gyms or universities, plays an active role in promoting or frustrating BPNs (Rodrigues et al., 2023). The BPN support offered by the subject’s significant others (e.g., parents, friends, instructors) is crucial for exercisers to feel integrated into the environment and to be able to regulate their motivation more autonomously (Deci and Ryan, 1987). This autonomous regulation tends to generate positive behavioral consequences, such as greater frequency of physical exercise and a stronger intention to continue practicing. Although the relationship between contextual aspects and BPNs is well established in the results, CET also emphasizes that the cognitive evaluation of contextual aspects influences the regulation of motivation. In other words, the contextual determinants that support autonomous motivation are equally important (Ryan and Deci, 2017). It is therefore essential for exercise professionals to create a supportive environment, fostering positive relationships with practitioners. This support tends to increase BPN satisfaction, reduce frustration, and prevent abandonment or quitting behavior, leading individuals to more self-determined forms of motivation regulation.

### 4.2 Basic psychological needs

In the studies included in this systematic review, there was a tendency for BPN satisfaction to be positively and significantly associated with more autonomous forms of motivation (Rodrigues et al., 2020; Rodrigues et al., 2022; Ng et al., 2014), while a negative and significant association was established between them and controlled forms of motivation (Rodrigues et al., 2020; Ng et al., 2014). This finding is in line with Self-Determination Theory (SDT), which posits that autonomous regulations are favored by the satisfaction of basic psychological needs (Ryan and Deci, 2000). Regarding BPN frustration, there has been a consistent tendency for it to be positively and significantly associated with more controlled forms of motivation, and negatively and significantly associated with more autonomous and self-determined regulations (Rodrigues et al., 2020). This trend of associations has already been suggested in various contexts and is one of the central assumptions of the theory of basic psychological needs (Bartholomew et al., 2011; Vansteenkiste and Ryan, 2013).

All the evidence is substantiated by the relationships established between BPNs and the ILO (Hagger and Chatzisarantis, 2008). According to the theory of BPNs, the locus of perceived causality refers to the degree to which behaviors have been internalized, reflecting the extent to which these behaviors meet goals valued by the individual. For example, when a person practices physical exercise with the intention of improving their physical condition, such behavior is more aligned with the promotion of BPN satisfaction (Ryan and Deci, 2000; Hagger and Chatzisarantis, 2008). In this context, BPN satisfaction facilitates the regulation of motivation in a more self-determined and autonomous way (interaction between BPN and OIT), especially when the environment supports this satisfaction (Teixeira et al., 2018). On the other hand, the frustration of these needs tends to predispose individuals to develop more controlled forms of motivation, which in turn is associated with negative behavioral consequences, such as less adherence to physical exercise (Bartholomew et al., 2009). In conclusion, SDT also conceptualized that the internalization of behavior is achieved through the satisfaction of BPNs and, consequently, the autonomous regulation of motivation seems to mediate the associations between the satisfaction of needs and behavioral consequences (Teixeira et al., 2018). At the same time, positive behavioral consequences in exercise contexts can, in turn, influence the way in which BPNs are satisfied, indirectly influencing motivational regulation (Schneider and Kwan, 2013).

### 4.3 Regulation of motivation

In the studies analyzed, positive and significant correlations were identified between more autonomous forms of regulation and variables such as enjoyment, intention, frequency of exercise and persistence. At the same time, there were negative and significant associations between more controlled forms of regulation and these same variables (Rodrigues et al., 2020; Rodrigues et al., 2022; Ng et al., 2013). In addition, there is a tendency for the more autonomous forms of regulation to correlate with both past and future behaviors of exercisers (Rodrigues et al., 2022). Specifically, integrated motivation tends to positively predict forms of positive affect, while amotivation and external regulation seem to negatively predict positive affect and exercise frequency, as well as positively predicting negative affect.

In this way, all the results discussed are in line with the ILO, in which BPNs play a central role in the process of internalizing behavior, being influenced by different channels of social interaction (Deci et al., 2013). Individuals naturally tend to adopt behaviors, norms or values that are transmitted to them by close sources, such as family, friends or exercise instructors (Deci et al., 2013). The more these values and norms are internalized, the more autonomous the individual’s conduct will be, and the social and environmental context is a determining factor in facilitating or inhibiting this internalization (Ryan and Connell, 1989; Howard et al., 2017). SDT proposes that behavior is regulated through mechanisms that determine the quality of motivation for an activity (e.g., physical exercise) (Deci and Ryan, 1985; Ryan and Deci, 2000). According to the macro theory, the individual’s involvement in the task is motivated according to their position along a motivational continuum, ranging from less self-determined to more self-determined forms (Wilson et al., 2003). In fact, the level of internalization can be expressed in six different forms of regulation along a motivational continuum, and these different forms directly influence the adoption of positive or negative behaviors. It is through the SDT motivational continuum that it is possible to carry out a refined analysis between the relationships established between the different types of regulation and behavioral consequences, in which more autonomous forms (e.g., intrinsic motivation) tend to associate and predict positive motivational consequences (e.g., persistence). On the other hand, controlled forms of motivation tend to be associated with negative motivational consequences, such as abandonment and subjective malaise (Ryan and Deci, 2000; Vallerand, 2007).

### 4.4 HMIEM: a sequential theoretical chain between micro models

Integrating the different micro-theories of SDT into a sequential chain gives us a holistic understanding of motivational processes in the context of physical exercise. There is theoretical and empirical evidence to support the causal chain between motivational variables and consequences (Rodrigues et al., 2023). In short, the context in which the individual is inserted (CET) can influence the way in which BPNs are satisfied or frustrated (TBPN), which in turn will influence the type of motivation regulation (OIT) that the individual will employ in relation to the behavior they want to adopt/maintain (Ryan and Deci, 2017; Vallerand and Toward, 1997; Rodrigues et al., 2023).

Regarding the vertical level of the HMIEM, in this systematic review all the studies analyzed were at the contextual level (life domains), represented essentially by the leisure situation (e.g., physical exercise) and the interpersonal relationships between instructor and practitioner (Vallerand and Toward, 1997). Within this hierarchical (contextual) level, this review has managed to fully cover the horizontal level (autonomous vs. controlled) presented by Vallerand (Vallerand and Toward, 1997). Thus, as conceptualized by Ryan and Deci (2017), we can see that the satisfaction and/or frustration of BPNs tends to be the result of how everyone perceives the contextual circumstances. In a practical sense, when an individual sees the exercise coach as a supportive figure, providing support and positive feedback, they are more likely to fulfil their BPNs (Rodrigues et al., 2019). Consequently, when the interpersonal behaviors between instructor and practitioner are identified as negligent and they are subject to constant negative feedback and pressure, the greater the likelihood of the latter being frustrated by BPNs.

Continuing to go through the motivational sequence, it shows how satisfaction/frustration of BPNs can have an impact on the regulation of motivation. Thus, there is a positive and significant relationship between BPN satisfaction and autonomous motivation (Vansteenkiste and Ryan, 2013). However, according to Ryan and Deci (2017) a positive and significant relationship can be expected between BPN frustration and more controlled forms of motivation. In fact, the results of this systematic review meet these assumptions, with some studies empirically demonstrating these theoretical relationships (Rodrigues et al., 2020; Rodrigues et al., 2022; Ng et al., 2013). Finally, the motivational sequence recognizes that the regulation of motivation can be related to different behavioral consequences, be they more behavioral, cognitive or emotional (Deci and Ryan, 1985; Ryan and Deci, 2017; Vallerand and Toward, 1997). Autonomous motivation, driven by the satisfaction of BPNs, tends to be associated with positive behavioral consequences, as presented in the results section of this systematic review (e.g., positive association with exercise frequency). Controlled motivation, on the other hand, is negatively related to positive behavioral consequences, leading to behaviors such as abandoning or giving up physical exercise (Silva et al., 2011).

### 4.5 Limitations and future directions

This literature review reveals several limitations that deserve attention in future research in the field of motivation in physical exercise. Firstly, about assessing the methodological quality of studies, the application of the Downs and Black Scale (Downs and Black, 1998) is pertinent. However, we suggest a mixed approach using two different scales: one for cross-sectional studies and the other for longitudinal studies. This strategy will allow for greater consistency in the assessment of methodological qualities, ensuring that the particularities of each type of study are adequately considered.

Secondly, the issue of bibliographical research stands out. Although many studies use the HMIEM model as a theoretical reference, it was observed that this information is not always explicit in the titles, abstracts, or keywords of the articles. This absence may have led to the inadvertent exclusion of relevant studies during the review. Furthermore, the exclusion of gray literature and articles written in other languages limits the scope of the results, since potential studies were excluded from this study, suggesting the need for a more inclusive and diverse search in future reviews.

A third limitation refers to the nature of the studies analyzed, all of which are cross-sectional and contextual. Only one longitudinal study was identified which investigated the relationships proposed by HMIEM over time. It is therefore recommended that future research not only consider longitudinal approaches but also explore other hierarchical levels of the model. Initially, these studies could adopt a cross-sectional approach and then move on to a multi-level longitudinal approach. This would make it possible to understand how different hierarchical levels interact in the context of exercise, providing a more integrated and dynamic view of the motivational process.

Another limitation identified concerns the variety of instruments used to analyze the variables. The heterogeneity of methods makes it difficult to compare results between studies, creating barriers to building accumulated and coherent knowledge on the subject. Future studies should therefore prioritize the use of the same instruments, provided they have been validated and translated for the specific cultural context, to facilitate the interpretation and cross-checking of data between different studies. A practical suggestion would be to develop and publish a guiding article for the scientific community, recommending the most suitable instruments for analyzing the variables in the HMIEM model. To achieve this, we would recommend a collaboration or creation of a panel of experts, in which a systematic review study with meta-analysis would be carried out to understand the best measurement instrument(s) for each variable. This would contribute to greater methodological standardization and, consequently, to the robustness of the conclusions derived from these investigations. These future guidelines, if implemented, could help to overcome the limitations currently observed in the literature, contributing to the advancement of knowledge about motivation in physical exercise and to the practical application of theoretical models in this context.

### 4.6 Practical implications

The HMIEM model has various practical implications for physical exercise, which are important for encouraging healthy and sustainable behaviors among exercisers. It has been observed that the HMIEM variables interact dynamically at the contextual level, and when properly respected and supported, they result in numerous behavioral benefits for exercisers. These interactions emphasize the importance of creating environments that support the satisfaction of basic psychological needs, promoting intrinsic motivation and longer-lasting adherence to exercise.

For political decision-makers, they have a huge responsibility to continue to support/create the conditions for physical exercise to be easily accessible to everyone. Strategies that can be implemented to facilitate this misstime and, for example, include financially helping some gyms and health clubs to reduce some of the costs associated with municipal services, promoting some events in municipalities to increase the level of physical activity through pleasurable activities that allow those involved to feel more motivated and to persist over time, and guaranteeing clean and attractive environments for safe and healthy physical exercise.

Gyms and fitness centers, in addition to coaches, have a significant responsibility in creating environments that promote enjoyment and ongoing physical exercise. These spaces should invest in continuous training for their staff, teaching them the benefits of using techniques and tools to measure the factors that affect consistent physical exercise. The relationship between the physical environment and the instructors should be structured to facilitate learning new methods and keeping practices up to date. This ensures that the gym is not only a place for physical exercise but also promotes long-term health and well-being.

In this sense, exercise instructors play a crucial role. They should create supportive environments, using positive feedback strategies and other motivational techniques. Interaction with clients should be based on encouragement and valuing individual efforts, which contributes to increasing the pleasure associated with physical exercise. This increase in pleasure, in turn, is directly related to higher levels of persistence, since exercisers tend to continue when they feel competent and supported.

The focus should be on the exerciser when developing strategies. It is crucial to value each individual and tailor interventions to combat low adherence to physical exercise and reduce resistance to gyms. The potential of gyms to significantly contribute to public health depends on their ability to offer more than just services. They should provide experiences that promote the overall well-being of their clients.

In short, the practical application of the guidelines derived from HMIEM in the context of physical exercise can transform the experience of practitioners, promoting greater adherence to physical exercise and, consequently, contributing to a better quality of life. This requires an integrated approach, where coaches, gyms, and practitioners work together to create motivating and sustainable environments.

## 5 Conclusion

This systematic review aimed to analyze the current state of the HMIEM model in the context of physical exercise, especially in gym and fitness environments. The results of this systematic review obtained through a rigorous methodological design indicate that supportive social factors are strongly associated with BPN satisfaction, autonomous motivation and exercise adherence. In contrast, controlling social factors showed negative associations with these variables, often resulting in abandonment of the practice.

The analysis reinforces the importance of BPN satisfaction and frustration as central elements in understanding the motivation and behavior of exercisers. Autonomous motivation was consistently related to positive behavioral results, while controlled motivation was associated with abandonment behavior. Thus, the creation of environments that favor autonomous motivation is essential to promote lasting adherence to exercise.

We conclude that the positive alignment between the variables of the HMIEM model, sustained by a supportive environment, is fundamental to adherence and maintenance of physical exercise behavior. These results offer valuable guidelines for implementing more effective strategies in gyms and fitness centers to promote a more active and healthier lifestyle. Thus, an exercise prescription focused on the individual, by satisfying their needs, will allow them to regulate their motivation in a more intrinsic way and end up persisting in physical exercise.
